# Ambient Temperature and Suicide Risk in Thailand: Evidence from Chiang Mai and Bangkok Provinces

**DOI:** 10.1021/envhealth.4c00153

**Published:** 2025-02-17

**Authors:** Ramita Thawonmas, Yoonhee Kim, Masahiro Hashizume

**Affiliations:** † School of Tropical Medicine and Global Health, Nagasaki University, Nagasaki 852-8523, Japan; ‡ Department of Global Environmental Health, Graduate School of Medicine, The University of Tokyo, Bunkyo, Tokyo 113-8654, Japan; § Department of Global Health Policy, Graduate School of Medicine, The University of Tokyo, Bunkyo, Tokyo 113-8654, Japan

**Keywords:** Ambient Temperature, Suicide Risk, Attributable Fraction, Mortality Burden, Thailand, Chiang Mai, Bangkok

## Abstract

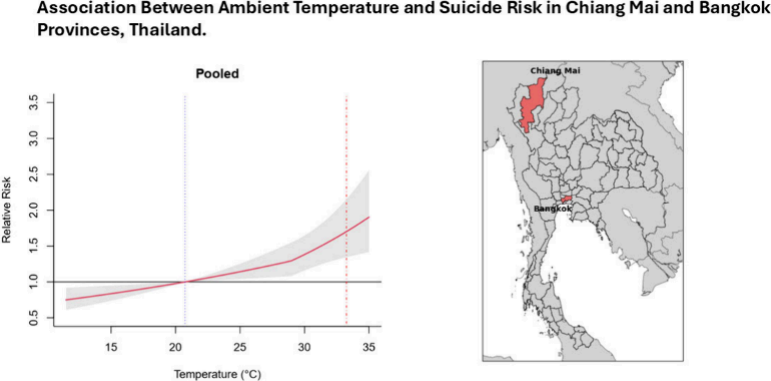

Suicide is a critical public health issue with rates varying across regions and demographic groups. Recent evidence suggests that ambient temperature may influence suicide risk. This study examines the association between temperature and suicide in Thailand’s tropical climate, focusing on Chiang Mai and Bangkok provinces, and quantifies the attributable burden. Daily suicide and meteorological data from 2002 to 2021 were analyzed using a time-stratified case-crossover approach with a distributed lag nonlinear model, adjusted for relative humidity. Province-specific estimates were pooled through a multivariate meta-regression model. The study found a positive, mostly linear association between temperature and suicide risk, with a relative risk (RR) of 1.70 (95% CI: 1.35, 2.15) across the temperature range. Approximately 24.61% of suicides were attributable to temperature, with 12.05% due to hot temperatures above the 66th percentile. The pooled attributable fractions were higher in the 0–64 age group compared to those aged ≥65, while differences between sexes were not statistically significant. This study highlights the significant association between higher ambient temperatures and increased suicide risks in Thailand, emphasizing the need to integrate climate considerations into mental health and suicide prevention policies. Further research across diverse climatic zones is essential for understanding climate influences on mental health globally.

## Introduction

Suicide is a major public health concern worldwide. More than 700,000 suicides occur every year globally, and suicide was the fourth leading cause of death in people aged 15–29 years.^1^ The global age-standardized suicide rate was estimated to be 9.0 per 100,000 population in 2019.^1^ Globally, the age-standardized suicide rate in 2019 was higher in males (12.6 per 100,000) than in females (5.4 per 100,000).^1^ Reduction of suicide mortality is one of the key priorities of the World Health Organization (WHO) and is included in WHO’s 13th General Programme of Work 2019–2023 and in the WHO Mental Health Action Plan 2013–2020, extended until 2030.^1^ There are also regional variations in suicide rates, with the majority (77%) of suicide deaths occurring in low-and middle-income countries.^1^

Thailand, situated in Southeast Asia, has one of the highest suicide rates in the region.^2^ In 2020, the total suicide rate was 7.8 persons per 100,000 population.^3^ Around 80% of completed suicides between 2013 and 2019 in Thailand involved men, with an average age of 45.37 years. Overall, there has been an increasing trend in suicides between 2013 to 2019 in Thailand.^4^

Suicide is a complex phenomenon with multiple interrelated risk factors, ranging from biological, psychological, and social factors.^5−7^ Recent studies have suggested that environmental factors, such as ambient temperature, may also play a role in triggering suicide, with an increased risk of suicide associated with higher temperatures.^8−12^ For instance, a meta-analysis found a relative risk (RR) of 1.016 (95% Confidence Interval [CI]:1.013, 1.019) for suicides and suicide attempts per 1 °C increase in ambient temperature.^13^ A multicountry study also found an association between higher ambient temperature and heightened suicide risk, with the RR of 1.33 (95% CI: 1.30, 1.36) for highest risk compared to the risk at the first percentile of temperature.^8^ The study found a clear nonlinear association in Japan, South Korea, and Taiwan, China, and a linear association in Western countries such as Canada, Spain, Switzerland, the UK, and the United States, while the association in Southeast Asian countries, namely the Philippines and Vietnam, was unclear with wide confidence intervals, potentially due to low numbers of suicide cases.^8^

The potential biological and psychosocial mechanisms linking temperature and suicide are complex and multifactorial. Rising temperatures may exacerbate existing mental health issues by triggering disruptions in neural activity, such as the overstimulation of heat-sensitive areas in the brain, which could aggravate anxiety and depressive symptoms.^14,15^ High temperatures have also been associated with increased sleep disturbances, which are closely linked to mental health and suicide risk.^16,17^ Additionally, serotonin, a neurotransmitter tied to mood regulation and impulse control, may be influenced by temperature fluctuations. Some studies suggest that low serotonin levels, possibly affected by higher temperatures, can heighten impulsivity and aggressive tendencies, potentially increasing the risk of suicide.^18,19^

Most of the existing evidence on the temperature-suicide association comes from high-income countries.^13^ To date, there have been no studies investigating this association in Thailand. Since Thailand is located within the tropics, and places with tropical climates are projected to experience higher health impacts from climate change,^20^ it is important to investigate the association to understand the temperature-related health effects to facilitate planning for public health interventions. In addition, limited research has quantified the attributable burden of suicide due to temperature in terms of attributable numbers or attributable fractions, with most evidence coming from China.^21,22^ Quantifying the actual impact is also valuable for planning public health interventions.^23^

The objective of this study was to investigate the association between temperature and suicide in the tropical context of Thailand, using daily time series data from Chiang Mai and Bangkok provinces. Additionally, the study aimed to quantify the suicide mortality burden attributable to temperature.

The study areas were Chiang Mai Province and Bangkok Province (Figure S1), which are among the most populous provinces in Thailand. Chiang Mai Province is located in the north of Thailand and is the largest province in northern Thailand. Bangkok is the capital of Thailand, located in central Thailand. Although both provinces have tropical climates with hot, wet, and cool seasons, Chiang Mai is generally colder and less humid.^24^ The suicide rate in Chiang Mai Province (population: 1,789,385 in 2021), which contains Chiang Mai city, the largest urban center in the northern region of Thailand, was 14.7 per 100,000 population, almost twice as high as the country’s overall rate.^3,25^ The suicide rate of the most populous Bangkok Province (population: 5,527,994 in 2021), was 4.3 per 100,000 population in 2020.^3^

## Materials and Methods

### Data

Daily suicide data from January 1, 2002 to December 31, 2021 for Chiang Mai Province and Bangkok Province were obtained from the Ministry of Public Health, Thailand. The data set includes all types of suicide, classified according to the International Classification of Disease, 10th revision (ICD-10)^26^ using the codes X60–X84, which encompass various suicide methods. The suicide data were reclassified by sex and age (0–64 years, ≥ 65 years old) for subgroup analysis. Daily meteorological data including minimum and maximum temperature and relative humidity were also obtained from the Thai Meteorological Department for the same period. For Bangkok, weather data were averaged from four available weather stations (stations 455201, 455203, 455301, and 455601), while in Chiang Mai, data were used from one of the two available stations (station 327501). The other station (station 327202), located in a remote mountainous area at an altitude of 1,400 m above sea level, was excluded as it was unlikely to represent the general weather conditions in Chiang Mai. This selection approach aligns with that used in a previous study.^27^ Daily maximum and minimum temperatures were averaged to obtain the mean temperature, representing the exposure throughout the day.

### Statistical Analyses

#### Estimation of Temperature–Suicide Association

A time-stratified case-crossover analysis was conducted to assess the association between ambient temperature and suicide for each province,^28^ using a two-stage approach. Specifically, in the first stage, a conditional Poisson regression considering overdispersion was fit. A stratum was designed as the interaction term of the year, calendar month, and day of the week. Each case was matched to several controls on the same day of the week in the same month and year. Strata without suicide events were excluded. By design, the case-crossover design already adjusted for the long-term trend, seasonality, and day of the week, with the assumption that unmeasured confounding factors that vary over time remained constant within a stratum.^28^ The distributed lag nonlinear modeling framework (DLNM) was applied when modeling the association between temperature and suicide to allow for potential nonlinear exposure-response and delayed effects.^29^ Based on the quasi-Akaike Information Criteria (QAIC), a linear B-spline with one internal knot at the 50th percentiles of province-specific temperature distributions was used for the exposure-response association. A maximum lag of 2 days was chosen, using a constrained DLNM with strata,^30^ based on previous studies.^8,31−33^ In addition, the model was adjusted for the 4-day moving average of relative humidity (modeled as a natural cubic spline with 1 degree of freedom) and for public holidays (as a binary variable).

In the second stage, we combined the estimates specific to each province through a multivariate meta-regression model.^34^ The methodology for calculating the exposure-response association and the details of this multivariate meta-regression model are elaborated in a previously published paper.^12^ We incorporated the average temperature of each province as a meta-predictor in the multivariate meta-regression, using the restricted maximum likelihood method, to account for climatic differences between provinces in the pooled estimates.

#### Quantifying Attributable Suicide Fraction Due to Temperature

Maximum suicide temperature (MaxST), which is the temperature corresponding to the maximum risk of suicide, between the first and 99th percentile of temperature, was identified from the meta-analysis results. Minimum suicide temperature (MinST) corresponded to the temperature with the lowest risk of suicide between the first and 99th percentiles of temperature. The lag-cumulative RR was estimated for MaxST versus MinST. The MinST and MaxST across the two provinces were used to calculate the pooled RRs, while the province-specific MinST and MaxST were used to calculate the province-specific RRs.

Next, the attributable fraction was calculated to quantify the attributable burden of suicide due to temperature. The MinST was used as the reference for calculating the attributable fractions using the ’attrdl’ function in R, using a method described in detail in previous research.^23,35^ Briefly, the overall cumulative RR corresponding to each day’s temperature was used to compute the attributable number and attributable fraction in the next 2 days in each province. The total attributable number of suicides due to temperature (namely, temperatures greater than or less than MinST) was computed by summing the contributions from all days of the series. The total attributable fraction is the ratio of the total attributable number and the total number of suicide deaths. The temperature data were classified into three categories: cool, warm, and hot temperatures. Cool temperatures correspond to the 0th to 33rd percentiles, warm temperatures to the 34th to 66th percentiles, and hot temperatures to the 67th to 100th percentiles. These cut points were used for quantifying attributable fractions due to different temperature ranges. Classifying temperatures into categories allows for a clearer analysis of how different temperature ranges specifically contribute to suicide burden. Similar methodologies have been used in other studies^36,37^ to enhance the understanding of temperature-related health impacts. The empirical confidence intervals (eCIs) for the attributable fractions were calculated through Monte Carlo simulations.

### Subgroup Analyses

Subgroup analyses by sex (male, female) and age group (0–64 years and ≥65 years) were conducted. The RRs for subgroups were calculated using the MinST and MaxST for all suicides. The attributable fractions for subgroups were calculated by using the subgroup-specific MinST for each province as the reference. The statistical significance tests between the differences between sex-specific, age-specific, and province-specific estimates were tested by the following equation:^38^
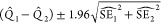
where  and  are the estimates for the two groups and  and  are the corresponding standard errors.

### Sensitivity Analyses

Sensitivity analyses were conducted to evaluate the robustness of the findings. These analyses involved altering the moving average of humidity to 2-day and 3-day moving averages, adjusting the maximum lag to 3, 6, 10, and 14 days, and changing the spline for the exposure-response lag to a natural cubic spline, quadratic B-spline, and linear function. Relative humidity averages were adjusted using a natural cubic spline with 1 degree of freedom.

All analyses were conducted with R (version 4.4.0) with packages “gnm” and “dlnm” for the time-stratified case-crossover analysis, and “mixmeta” for the multivariate regression.

## Results

Between 2002 and 2021, 8472 suicides were registered in Chiang Mai and Bangkok provinces combined (Table 1).

**Table 1:** Descriptive Statistics of Daily Suicide and Meteorological Variables in Chiang Mai and Bangkok Provinces and Both Provinces Combined, 2002–2021

	Chiang Mai				Bangkok				Combined			
	Total	Mean (SD)	Min	Max	Total	Mean (SD)	Min	Max	Total	Mean (SD)	Min	Max
Overall suicide	5000	0.7 (0.8)	0	6	3472	0.5 (0.7)	0	4	8472	0.6 (0.8)	0	6
Sex												
Male	4045 (80.9%)	0.6 (0.8)	0	6	2641 (76.1%)	0.4 (0.6)	0	4	6686 (78.9%)	0.5 (0.7)	0	6
Female	955 (19.1%)	0.1 (0.4)	0	3	831 (23.9%)	0.1 (0.3)	0	3	1786 (21.1%)	0.1 (0.4)	0	3
Age												
0–64	4361 (87.2%)	0.6 (0.8)	0	6	3097 (89.2%)	0.4 (0.7)	0	4	7458 (88.0%)	0.5 (0.7)	0	6
≥65	639 (12.8%)	0.1 (0.3)	0	3	375 (10.8%)	0.1 (0.2)	0	2	1014 (12.0%)	0.1 (0.3)	0	3
Mean temperature (°C)	-	27.3 (2.8)	11.1	35.1	-	29.6 (1.8)	18.5	34.7	-	28.5 (2.6)	11.1	35.2
Mean relative humidity (%)	-	71.2 (11.8)	36.0	99.0	-	71.2 (8.1)	45	96	-	71.2 (10.1)	36.0	99.0

Males accounted for 78.9% of suicides, and the age group 0–64 years accounted for 88.0%. The mean daily temperature was 28.5 °C, and the mean daily relative humidity was 71.2%. Table 1 shows the descriptive statistics for daily suicide deaths, mean temperature, and relative humidity for each province.

The daily mean temperature peaked in April in both Chiang Mai and Bangkok and reached a trough in December through January (Figure S2). Seasonality in suicide was observed, with peaks in hot (March-May) and wet (June-October) seasons, and troughs in the cool (November-February) season (Figure S3). Figure S4 shows the decomposition analysis of daily suicides in Chiang Mai (Figure S4A) and Bangkok (Figure S4B) from 2002 to 2021. In Chiang Mai, suicide rates showed a downward trend from 2002 to 2009, stabilized from 2010 to 2018, rose in 2019, and decreased in 2021. In Bangkok, rates decreased from 2002 to 2005, rose in 2007, decreased until 2014, and rose from 2015.

**Figure 1:**
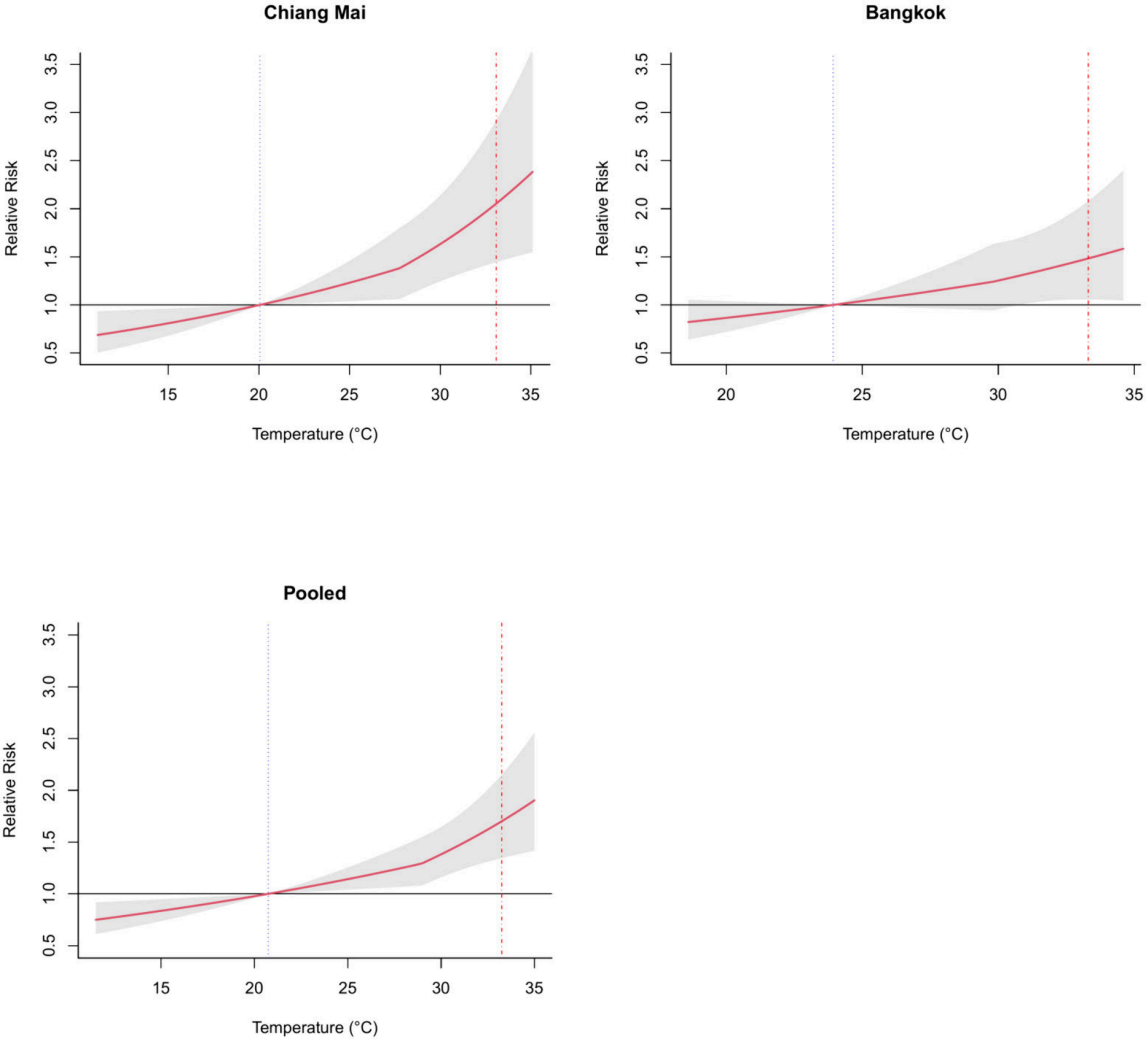
Overall cumulative relative risk curve for Chiang Mai, Bangkok, and both provinces (pooled). Temperature-suicide associations. Two vertical lines are the minimum suicide temperature (MinST) as a dotted line in blue and the maximum suicide temperature (MaxST) as a dash-dotted line in red. The MinST and MaxST for Chiang Mai were 20.1 and 33.1 °C, respectively. The MinST and MaxST for Bangkok were 23.9 and 33.3 °C, respectively. For both provinces, the MinST and MaxST corresponded to the first and 99th percentile of the temperature distribution, respectively. The MinST and MaxST across the two provinces were 20.7 and 33.3 °C respectively, which corresponded to the first and 99th percentile of the temperature distribution across the two provinces. The shaded areas are the 95% Confidence Interval.

Figure 1 shows the associations between temperature and suicide in Chiang Mai and Bangkok, and the pooled overall cumulative risk curve for both provinces, with a positive, mostly linear association, and increased risk at higher temperatures.

The MaxST and MinST were 33.1 and 20.1 °C in Chiang Mai, and 33.3 and 23.9 °C in Bangkok, representing the 99th and first percentiles, respectively. The RR for MaxST versus MinST was 2.05 (95% CI: 1.45, 2.92) in Chiang Mai and 1.48 (95% CI: 1.06, 2.08) in Bangkok, with a pooled RR of 1.70 (95% CI: 1.35, 2.15) (Table 2).

**Table 2:** Overall Cumulative Relative Risk and 95% Confidence Interval for Chiang Mai, Bangkok, and Both Provinces (Pooled)^a^

Group	Chiang Mai	Bangkok	Both Provinces (Pooled)
Overall	2.05 (1.45, 2.92)	1.48 (1.06, 2.08)	1.70 (1.35, 2.15)
Sex			
Male	1.80 (1.22, 2.67)	1.57 (1.07, 2.32)	1.64 (1.26, 2.13)
Female	3.44 (1.53, 7.73)	1.19 (0.59, 2.40)	1.98 (1.19, 3.29)
Age			
0–64	2.28 (1.57, 3.32)	1.55 (1.09, 2.21)	1.83 (1.42, 2.34)
≥65	1.04 (0.39, 2.74)	0.95 (0.32, 2.82)	1.02 (0.54, 1.94)

a The overall cumulative relative risk is the relative risk for maximum suicide temperature (MaxST) versus minimum suicide temperature (MinST). The MinST and MaxST for Chiang Mai were 20.1 and 33.1 °C respectively. The MinST and MaxST for Bangkok were 23.9 and 33.3 °C respectively. For both provinces, the MinST and MaxST corresponded to the first and 99th percentile of the temperature distribution, respectively. The MinST and MaxST across the two provinces were 20.7 and 33.3 °C respectively, which corresponded to the first and 99th percentile of the temperature distribution across the two provinces.

**Figure 2:**
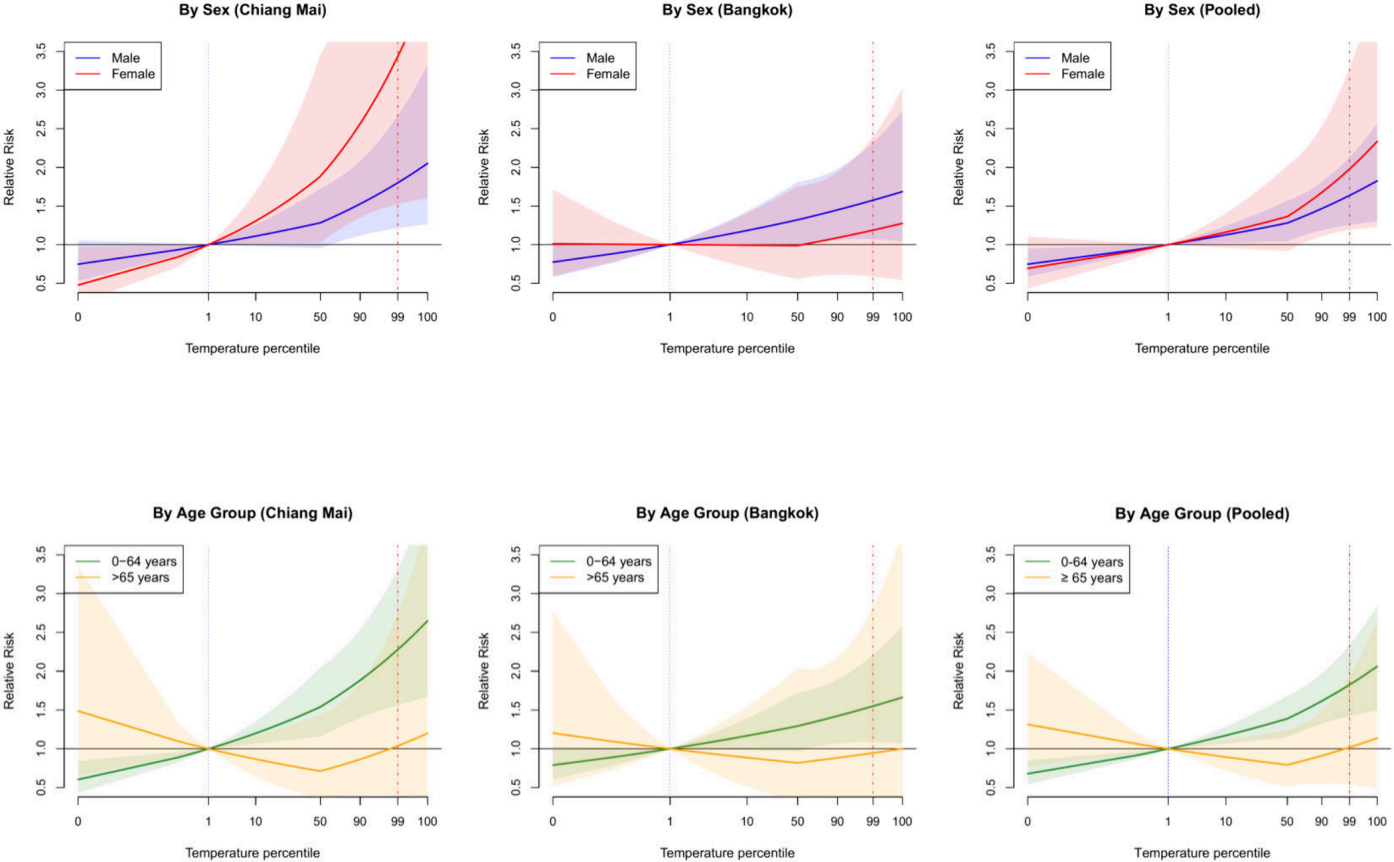
Overall cumulative relative risk curve by sex and age subgroups for Chiang Mai, Bangkok, and both provinces (pooled). Temperature–suicide associations by sex and by age for Chiang Mai, Bangkok, and both provinces combined (pooled). Two vertical lines indicate the minimum suicide temperature percentile as a dotted line in blue and the maximum suicide temperature percentile as a dash-dotted line in red. The shaded areas represent the 95% Confidence Intervals.

Figure 2 presents the cumulative RR curves for subgroups, with RRs and 95% CIs in Table 2.

Positive, mostly linear patterns were observed across subgroups, except for those aged ≥65 years. In Chiang Mai, females had a higher RR (3.44, 95% CI: 1.53, 7.73) than males (1.80, 95% CI: 1.22, 2.67), although the confidence intervals largely overlapped. Conversely, in Bangkok, males had a higher RR (1.57, 95% CI: 1.07, 2.32) than females (1.19, 95% CI: 0.59, 2.40), with uncertainty for females. In Chiang Mai, the RR for those aged 0–64 years was 2.28 (95% CI: 1.57, 3.32), higher than for those aged ≥65 years (1.04, 95% CI: 0.39, 2.74). Similarly, in Bangkok, the RR for those aged 0–64 years was 1.55 (95% CI: 1.09, 2.21), higher than for those aged ≥65 years (0.95, 95% CI: 0.32, 2.82), with uncertainty for the older age group.

Figure 2 also displays the pooled overall cumulative RR curves for sex and age subgroups, and Table 2 presents the corresponding RRs and 95% CIs. Positive, mostly linear patterns were seen compared to overall suicides, except for those aged ≥65 years. The pooled RR for females (1.98, 95% CI: 1.19, 3.29) was higher than that for males (1.64, 95% CI: 1.26, 2.13), and the RR for those aged 0–64 years (1.83, 95% CI: 1.42, 2.34) was higher than for those aged ≥65 years (1.02, 95% CI: 0.54, 1.94), although the confidence intervals largely overlapped.

Table 3 shows the attributable fractions of suicide due to temperature for the overall group and sex and age subgroups in Chiang Mai and Bangkok.

**Table 3:** Attributable Fraction of Suicide Due to Temperature in Chiang Mai, Bangkok, and Both Provinces (Pooled)^a^

Group	Province	Total % (95% CI)	Cool % (95% CI)	Warm % (95% CI)	Hot % (95% CI)
Overall	Chiang Mai	27.97 (11.33, 42.66)	4.73 (1.04, 8.05)	9.48 (3.03, 14.73)	14.21 (7.86, 19.58)
	Bangkok	19.33 (−1.07, 35.90)	4.11 (−1.25, 8.38)	6.57 (−1.11, 12.76)	8.81 (0.56, 14.98)
	Pooled	24.61 (10.78, 34.68)	4.55 (1.63, 7.20)	8.35 (2.76, 12.49)	12.05 (6.32, 16.10)
Male	Chiang Mai	22.89 (2.03, 40.02)	3.71 (−0.80, 7.21)	7.66 (−0.87, 13.91)	11.91 (3.46, 18.10)
	Bangkok	23.48 (−0.86, 39.60)	5.12 (−0.58, 9.46)	8.12 (−1.36, 14.15)	10.43 (1.68, 17.14)
	Pooled	23.32 (8.13, 35.79)	4.33 (0.64, 7.19)	7.91 (1.66, 12.74)	11.38 (5.22, 16.00)
Female	Chiang Mai	45.07 (10.81, 65.19)	8.73 (0.23, 14.44)	15.52 (0.88, 22.87)	21.48 (8.80, 28.31)
	Bangkok	2.98 (−10.92, 13.79)	0.12 (−6.99, 5.42)	0.27 (−0.82, 1.33)	2.60 (−7.63, 9.00)
	Pooled	25.62 (8.90, 37.52)	4.78 (−1.22, 8.93)	8.46 (1.34, 12.67)	12.74 (4.89, 17.57)
0–64	Chiang Mai	34.01 (16.24, 46.10)	6.19 (2.41, 9.32)	11.90 (5.19, 17.15)	16.47 (9.43, 21.39)
	Bangkok	22.07 (−0.08, 38.54)	4.82 (0.04, 9.19)	7.64 (−0.55, 13.81)	9.83 (1.68, 16.03)
	Pooled	29.26 (16.38, 39.18)	5.70 (2.35, 8.46)	10.21 (5.06, 14.36)	13.77 (8.69, 17.89)
≥65	Chiang Mai	10.71 (−8.37, 23.03)	4.46 (−5.33, 11.35)	0.93 (−0.41, 2.36)	5.43 (−8.07, 13.87)
	Bangkok	4.45 (−19.95, 20.79)	1.96 (−9.93, 8.83)	0.33 (−1.17, 1.61)	2.17 (−15.10, 12.65)
	Pooled	8.58 (−5.35, 19.36)	3.58 (−3.99, 9.08)	0.77 (−0.32, 1.82)	4.31 (−5.51, 10.68)

a Attributable fraction % (95% empirical CI) computed as total fraction and as separate components for cool (0th–33rd), warm (34th–66th), and hot (67th–100th). The attributable fractions for the overall group were calculated using the province-specific minimum suicide temperature (MinST), which was 20.1 °C (first percentile temperature) for Chiang Mai province and 23.9 °C (first percentile temperature) for Bangkok province. The attributable fractions for subgroups were calculated using the subgroup-specific MinST for each province as the reference. Subgroups, except for females in Bangkok and people aged ≥65 years in both provinces, had the same MinST as the overall groups. MinST of 29.8 °C (50th percentile temperature) was used to calculate the attributable fractions for females in Bangkok. MinST of 27.8 °C (50th percentile temperature) and 29.8 °C (50th percentile temperature) were used to calculate the attributable fractions for the ≥65 years group in Chiang Mai and Bangkok, respectively.

In Chiang Mai, 27.97% (95% empirical CI (eCI): 11.33, 42.66) of suicides were attributable to temperature, while in Bangkok, 19.33% (95% eCI: −1.07, 35.90) were attributable, though this estimate was uncertain.

**Figure 3:**
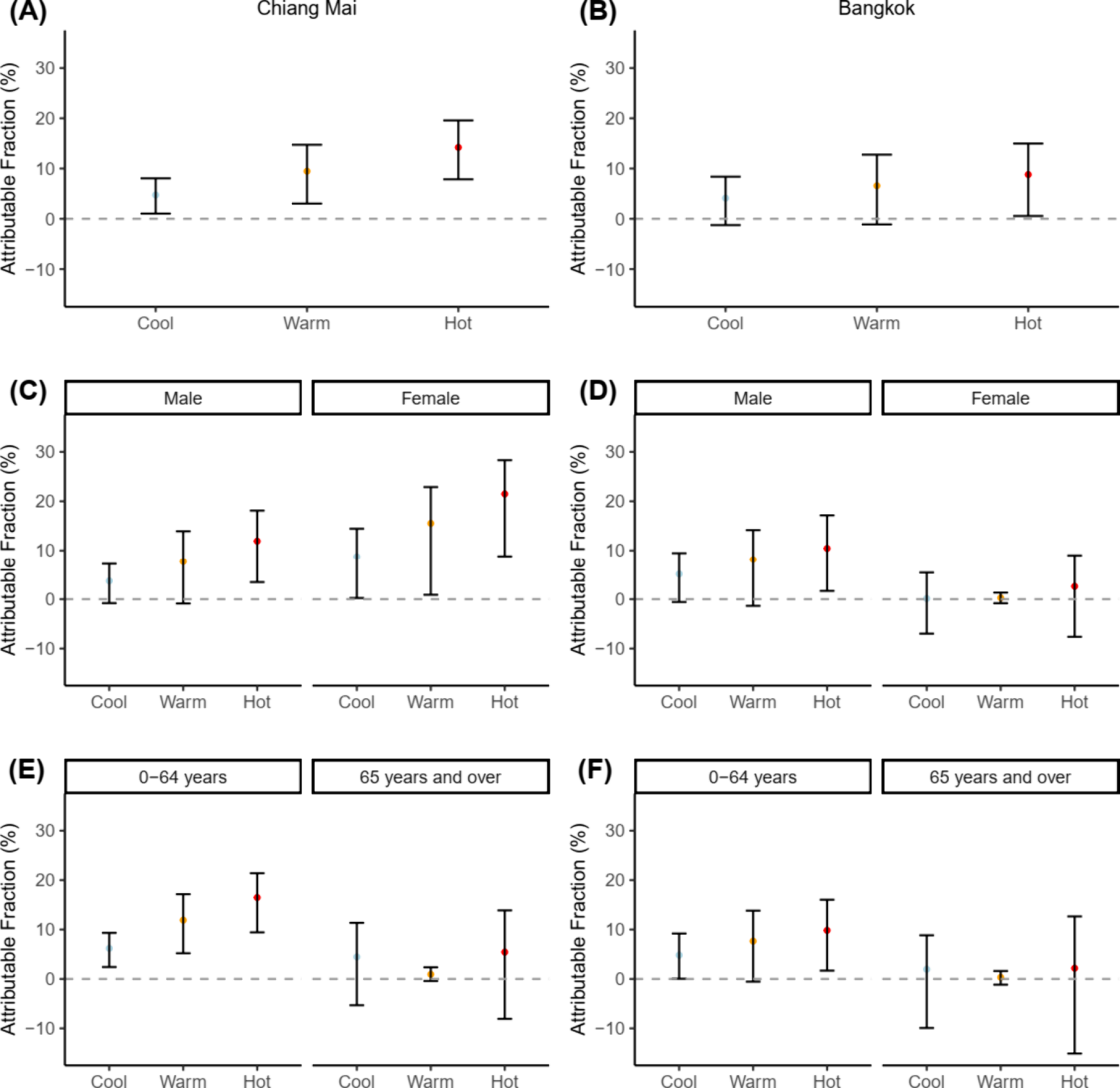
Attributable burden of suicide due to cool (0th–33rd percentile), warm (34th–66th), and hot (67th–100th) temperatures. (A) Attributable fraction (%) and 95% empirical CI for the overall group in Chiang Mai. (B) Attributable fraction (%) and 95% empirical CI for the overall group in Bangkok. (C) Attributable fraction and 95% empirical CI for the male and female in Chiang Mai. (D) Attributable fraction and 95% empirical CI for the male and female in Bangkok. (E) Attributable fraction and 95% empirical CI for the group aged 0–64 years and ≥65 years in Chiang Mai. (F) Attributable fraction and 95% empirical CI for the group aged 0–64 years and ≥65 years in Bangkok. The attributable fractions for the overall groups were calculated using the province-specific minimum suicide temperature (MinST), which was 20.1 °C (first percentile temperature) and 23.9 °C (first percentile temperature) for Chiang Mai and Bangkok provinces respectively. The attributable fractions for subgroups were calculated by using the subgroup-specific MinST for each province as the reference. Subgroups except for females in Bangkok and people aged ≥65 years in both provinces had the same MinST as the overall groups. MinST of 29.8 °C (50th percentile temperature) was used to calculate the attributable fractions for females in Bangkok. MinST of 27.8 °C (50th percentile temperature) and 29.8 °C (50th percentile temperature) were used to calculate the attributable fractions for ≥65 years group in Chiang Mai and Bangkok respectively.

Hot temperatures had the highest attributable fractions in Chiang Mai (14.21%, 95% eCI: 7.86, 19.58) and Bangkok 8.81%, 95% eCI: 0.56, 14.98) for overall suicides (Figure 3).

Subgroup analyses showed 22.89% (95% eCI: 2.03, 40.02) and 45.07% (95% eCI: 10.81, 65.19) of suicides among males and females in Chiang Mai were due to temperature, respectively. In Bangkok, these fractions were 23.48% (95% eCI: −0.86, 39.60) for males and 2.98% (95% eCI: −10.92, 13.79) for females, both uncertain. The 0–64 years age group had fractions of 34.01% (95% eCI: 16.24, 46.10) in Chiang Mai and 22.07% (95% eCI: −0.08, 38.54) in Bangkok, with Bangkok’s estimate uncertain. For the ≥65 years group, the fractions were 10.71% (95% eCI: −8.37, 23.03) in Chiang Mai and 4.45% (95% eCI: −19.95, 20.79) in Bangkok, both uncertain.

Similar patterns were seen for sex- and age-specific attributable fractions, with hot temperatures having the highest attributable fractions when estimates were not uncertain (Figure 3).

**Figure 4:**
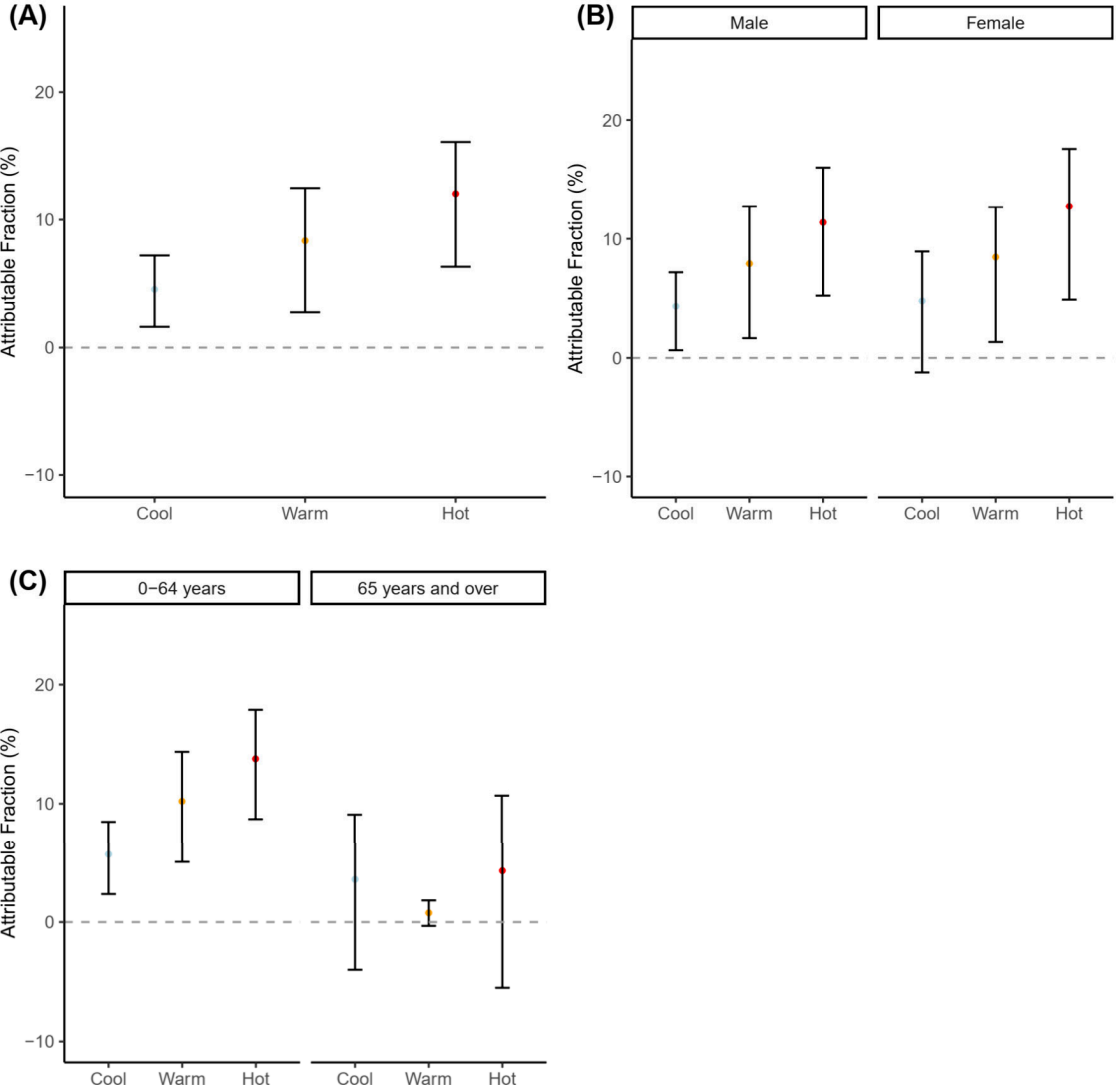
Attributable burden of suicide due to cool (0th–33rd percentile), warm (34th–66th), and hot (67th–100th) temperatures in Chiang Mai and Bangkok provinces (Pooled). (A) Pooled attributable fraction (%) and 95% empirical CI for the overall group. (B) Pooled attributable fraction and 95% empirical CI for the male and female. (C) Pooled attributable fraction and 95% empirical CI for the group aged 0–64 years and ≥65 years. The pooled attributable fractions for the overall groups were calculated using the province-specific minimum suicide temperature (MinST), which was 20.1 °C (first percentile temperature) and 23.9 °C (first percentile temperature) for Chiang Mai and Bangkok provinces respectively. The attributable fractions for subgroups were calculated by using the subgroup-specific MinST for each province as the reference. Subgroups except for females in Bangkok and people aged ≥65 years in both provinces had the same MinST as the overall groups. MinST of 29.8 °C (50th percentile temperature) was used to calculate the attributable fractions for females in Bangkok. MinST of 27.8 °C (50th percentile temperature) and 29.8 °C (50th percentile temperature) were used to calculate the attributable fractions for ≥65 years group in Chiang Mai and Bangkok respectively.

Table 3 also shows the pooled attributable fractions of suicide due to temperature for the overall group, as well as the sex and age subgroups. Overall, 24.61% (95% eCI: 10.78, 34.68) of suicides were attributable to temperature. The attributable fractions for different temperature ranges and groups are also depicted in Figure 4.

Most suicides were attributable to hot (12.05%, 95% eCI: 6.32, 16.10) and warm (8.35%, 95% eCI: 2.76, 12.49) temperatures. Similar patterns were observed for the pooled sex-specific and age-specific attributable fractions due to different temperature ranges, with hot temperatures accounting for the highest attributable fractions.

Sex-specific and age-specific RRs and attributable fractions within each province were not statistically significant, except for the age-specific attributable fractions in Chiang Mai, where the 0–64 years age group (34.01%, 95% eCI: 16.24, 46.10) had a higher burden than the ≥65 years group (10.71%, 95% eCI: −8.37, 23.03). The difference between the overall province-specific RRs in Chiang Mai (2.05, 95% CI: 1.45, 2.92) and Bangkok (1.48, 95% CI: 1.06, 2.08) for all suicides, as well as the attributable fractions, was also not statistically significant. For the differences between pooled sex-specific and age-specific RRs and attributable fractions, only the differences between pooled age-specific attributable fractions were statistically significant, with a higher burden for the 0–64 years age group (29.26%, 95% eCI: 16.38, 39.18) compared to the ≥65 years group (8.58%, 95% eCI: −5.35, 19.36).

In Chiang Mai, the RR increased immediately on the day of exposure (lag 0 day) for hotter temperatures, while in Bangkok, the increase in RR at hotter temperatures occurred on lag 1 to lag 2 day as well (Figure S5).

Sensitivity analyses indicated that the estimates were robust for both locations, as the RR remained stable despite adjustments to model parameters, including changes to the moving average day for humidity, modifications to the lag duration, adjustments to a natural or quadratic B-spline, and substitution with a linear function (Figure S6). Extending the lags up to 14 days resulted in less precise estimates, as shown by wider confidence intervals.

## Discussion

Our findings suggest evidence for an association between higher temperatures and an increased risk of suicide in Thailand, with a particular focus on Chiang Mai and Bangkok provinces. The attributable fraction analysis revealed a higher burden for the 0–64 years age group compared to those aged ≥65 years. Furthermore, we found that approximately 24.61% of suicides could be attributed to temperature, with 12.05% attributable to hot temperatures.

Our pooled RR estimates, which demonstrate a significant association between higher temperatures and increased suicide risks in Thailand, align with similar studies conducted in various geographical and climatic contexts. For instance, a multicountry study found a significant positive association between temperature and suicide in Western countries and East Asia.^8^ Another study in Japan has found nonlinear association between ambient temperature and suicide risk.^12^ Similarly, research in the United States has demonstrated that higher temperatures are associated with increased suicide rates.^39^ Our study extends these findings into the context of a tropical climate, where such associations have been less explored. Our findings of nearly linear association in two provinces in Thailand contrast with a multicity study which found largely unclear associations in the tropics (Philippines and Vietnam) while finding nearly linear associations in the Western countries (Canada, Spain, Switzerland, the UK, and the United States and nonlinear associations in East Asia (Japan, South Korea, and Taiwan, China).^8^

The apparently higher overall cumulative RR for all suicides observed in Chiang Mai Province compared to Bangkok Province aligns with observations from Japan, where the strength of the temperature-suicide association was found to vary among the prefectures, with higher RRs in colder regions, possibly due to lower adaptation to hot temperatures among people living in colder climates.^12^ Although the difference in RRs between Chiang Mai and Bangkok was not statistically significant, Chiang Mai Province has a cooler climate compared to Bangkok, which may help explain the observed pattern. The observed difference in relative risk (RR) between Chiang Mai and Bangkok may be influenced by a combination of climatic, biological, and sociocultural factors. While the cooler climate in Chiang Mai may reduce acclimatization to heat, differences in urbanization and access to mental health services, as well as other sociocultural factors, may also play a role. Chiang Mai’s population may have less access to mental health resources compared to Bangkok, a major metropolitan area.^40^ For example, in 2021, Bangkok had 4.9 psychiatrists per 100,000 population while Chiang Mai province had 2.5 psychiatrists per 100,000 population.^40^ Additionally, differences in employment patterns, such as a higher prevalence of outdoor labor including in the agricultural sector in Chiang Mai,^41^ could contribute to increased exposure to high temperatures, thereby influencing suicide risk. Future research is warranted to better understand the interplay of environmental, biological, and sociocultural factors in influencing suicide risk.

Furthermore, our study’s findings of observed varied age-specific RRs and sex-specific RRs are echoed in previous research, which have similarly observed differences in the temperature-suicide association across sex and age groups, though the direction of these associations varies.^8,12,32,42^

The positive association between ambient temperature and suicide risk identified in our study aligns with existing global evidence suggesting that higher temperatures may exacerbate the risk of suicide.^8,12,43^ Our findings contribute to this body of knowledge by suggesting that this association may also be relevant in the tropical climate of Thailand, a context previously underrepresented in the literature. Although differences in RRs between age groups were not statistically significant, the observed trend of higher RRs for the 0–64 years age group, along with a statistically significant higher burden in this age group, suggests that occupational exposure to ambient temperatures, particularly in outdoor or non-air-conditioned environments, may play a role in modulating risk.^44^

There was no evidence of a difference in the association between males and females. This contrasts with several studies that found a greater RR among males, for example in Spain, the UK, Croatia, and Brazil,^8,45,46^ and it has been suggested that men may be more susceptible than women due to their higher engagement in outdoor jobs, which may lead to more frequent exposure to ambient temperatures.^47^ However, the effects of temperature on suicide risk may also depend on geographical location or cultural factors, as in other countries such as Japan, females appeared to be more susceptible.^8,12^

The percentage of suicides attributable to temperature in Thailand (24.61%) suggests that ambient temperature may be an important environmental stressor with a considerable public health impact. This may be particularly relevant for policy-making in tropical regions where climate change may further exacerbate temperature extremes.^20^

Comparison with previous studies on the attributable fractions of suicide due to temperature is challenging due to the limited number of such studies. Nonetheless, parallels can be drawn with existing research from China and Japan. For instance, a study in Shenzhen, China, found that the attributable fraction of emergency ambulance dispatches for suicide was 13.82% (95% eCI: −6.56, 28.15), although these estimates were uncertain.^9^ Similar to our findings of lower attributable fractions due to cooler temperatures, the study reported a lower attributable fraction due to cold temperatures (0.07%, 95% eCI: −1.23, 1.28) compared to hot temperatures (13.76%, 95% eCI: −6.10, 28.13).^9^ While the Chinese study categorized temperatures below and above the optimal temperature (the temperature with the lowest risk) as cold and hot, respectively,^9^ our study used a more detailed classification into cool, warm, and hot temperature ranges.

In Japan, a study analyzing data from 47 prefectures found that approximately 19.9% of suicides could be attributed to nonoptimal temperatures, with the highest fraction (9.9%) observed for warm temperatures (50th-90th percentile).^37^ The study highlighted that higher burdens were observed in females (23.7%), individuals aged 65 years and older (31.9%), and violent suicides (22.4%).^37^ These findings contrast with our results, which identified the highest attributable fraction of suicides due to hot temperatures (12.05%) in Thailand. This difference suggests that while warmer temperatures play a significant role in both contexts, the extremes of hot temperatures are particularly impactful in Thailand’s tropical climate.

The underlying mechanisms driving the association between temperature and suicide risk remain an area of active investigation. One leading hypothesis involves serotonin, a neurotransmitter known to modulate mood, aggression, and impulsivity, factors that are often implicated in suicidal behavior.^48^ Elevated ambient temperatures have been hypothesized to affect serotonergic neurotransmission, potentially exacerbating mood disorders, increasing impulsivity, and thereby potentially increasing the risk of suicide.^49^ Additionally, higher temperatures may also disrupt the body’s stress response system,^50^ further aggravating mental health conditions and possibly increasing vulnerability to suicidal thoughts and behaviors. Furthermore, disrupted sleep patterns, another factor closely linked to mental health issues,^51^ may serve as an intermediary; heat-induced sleep disturbances may exacerbate mental health conditions,^50^ thereby potentially amplifying the risk of suicide.

To our knowledge, this is the first investigation into the temperature-suicide association in Thailand, a tropical country where this association has not previously been explored. Another strength is the utilization of a 20-year comprehensive data set of suicide rates in two of the most populous Thai provinces. The robust methodological approach, employing a time-stratified case-crossover design, provided a control for long-term trends, seasonality, and day-of-the-week effects, minimizing potential confounding factors that could influence the study outcomes. Our study also contributes insights into the health impacts of ambient temperature by quantifying the attributable fractions of suicide due to temperature, contributing to the currently limited evidence in this field.

Several limitations of our study must be acknowledged. First, the ecological nature of the study means that the results are based on population-level data, which precludes the establishment of causality for individual cases. Additionally, while we averaged data from four weather stations in Bangkok to capture temperature variability, we used a single weather station in Chiang Mai (station 327501) due to the other station’s remote mountainous location at 1,400 m above sea level, which may not reflect the general weather conditions of the province. This approach aligns with a prior study in Chiang Mai,^27^ yet it may still limit our ability to fully capture temperature variability within the province. Moreover, the use of ambient temperature data from weather stations may not accurately reflect the exposure experienced by individuals, potentially leading to Berkson-type measurement errors.^52^ The study’s reliance on death certificate data for suicide classification, while standardized through ICD-10 codes, also raises the possibility of misclassification due to reporting inaccuracies and possible underreporting of suicides due to stigma.^53^ In addition, the subgroup analysis for specific demographic groups, such as females, and older adults (≥65 years), yielded uncertain estimates. This uncertainty likely arises from the relatively low number of suicide cases within these subgroups, reflecting data limitations. Finally, we did not adjust for air pollution in our analysis. While this may be a potential confounder, evidence from some temperature-mortality studies suggests that the association between temperature and mortality remains robust even after controlling for air pollutants.^54−59^

Our study highlights a clear association between higher temperatures and increased suicide risks, underscoring the importance of integrating temperature considerations into public health strategies. The attributable fraction analysis findings indicate that approximately one-quarter of suicides in our study regions may be associated with temperature, with about half of this burden specifically linked to hot temperatures above the 66th percentile. These findings suggest the considerable potential burden of temperature-related suicide, particularly as climate change is expected to amplify temperature extremes. Our results suggest the importance of incorporating mental health and suicide prevention strategies into broader climate change adaptation policies. This integration could complement existing interventions, such as heat-health warning systems^60^ and mental health support campaigns during warmer periods, to help address the potential increase in temperature-related suicide burden in the future. Furthermore, the variability observed in Chiang Mai and Bangkok provinces emphasizes the necessity for broader research across different climates to understand the global dynamics of temperature and suicide risk. This would help in developing nuanced public health responses that consider the interplay of environmental, sociocultural, and economic factors in suicide prevention efforts, particularly under the evolving challenges posed by climate change.

## Conclusions

This study elucidates the significant link between ambient temperature and suicide risk within Thailand’s tropical setting, highlighting a particularly higher temperature-related burden in younger populations. These findings contribute to the evidence base on environmental factors affecting mental health and underscore the need for climate-informed suicide prevention and mental health policies. The age-specific variations in the attributable fraction of suicides suggest that younger populations may be more vulnerable to temperature-related impacts, emphasizing the necessity for tailored public health interventions. Future research should prioritize examining temperature-suicide associations across other tropical regions, assessing the effectiveness of interventions such as heat-health warning systems and mental health support campaigns during warmer periods, and exploring how these efforts can be integrated into broader climate adaptation strategies. Such studies will be essential for developing evidence-based, region-specific strategies to address the challenges posed by climate change and its impacts on mental health.

## Supporting Information Available

Figure S1, Locations of Chiang Mai and Bangkok, Thailand; Figure S2, Time series of daily mean temperature in Chiang Mai and Bangkok; Figure S3, Seasonal suicide trends in Chiang Mai and Bangkok; Figure S4, Decomposition of additive time series in Chiang Mai and Bangkok; Figure S5, 3D plot showing the estimated exposure–lag–response association between temperature and suicide in Chiang Mai and Bangkok; Figure S6, Sensitivity analysis: Relative risks with 95% CIs (vertical bars) (PDF)
