# Supplementary material for: Ambient Temperature and Suicide Risk in Thailand: Evidence from Chiang Mai and Bangkok Provinces
Source: Environ Health (Wash). 2025 Feb 17;3(5):560–70. doi: 10.1021/envhealth.4c00153 (PMC12090010; doi:10.1021/envhealth.4c00153)
Supplement: Supplementary file 1 — eh4c00153_si_001.pdf [file eh4c00153_si_001.pdf]

# Supporting Information

## Ambient Temperature and Suicide Risk in Thailand: Evidence from Chiang Mai and Bangkok Provinces

Ramita Thawonmas[1]*, Yoonhee Kim[2], Masahiro Hashizume[3]

[1]School of Tropical Medicine and Global Health, Nagasaki University, Nagasaki, Nagasaki 852-8523, Japan.

ramita.thawonmas@nagasaki-u.ac.jp

[2]Department of Global Environmental Health, Graduate School of Medicine, The University of Tokyo, Bunkyo, Tokyo 113-8654, Japan

[3]Department of Global Health Policy, Graduate School of Medicine, The University of Tokyo, Bunkyo, Tokyo 113-8654, Japan

## Table of Contents

**Figure S1.** Locations of Chiang Mai and Bangkok, Thailand.

**Figure S2.** Time series of daily mean temperature in Chiang Mai and Bangkok.

**Figure S3.** Seasonal suicide trends in Chiang Mai and Bangkok.

**Figure S4.** Decomposition of additive time series of suicide in Chiang Mai and Bangkok.

**Figure S5.** 3D plot showing the estimated exposure–lag–response association between temperature and suicide in Chiang Mai and Bangkok.

**Figure S6.** Sensitivity analysis: Relative risks with 95% CIs (vertical bars).

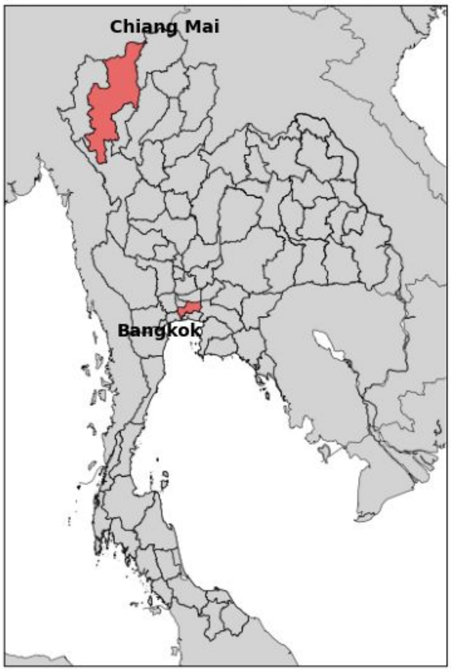


**Figure S1. Locations of Chiang Mai and Bangkok, Thailand.**

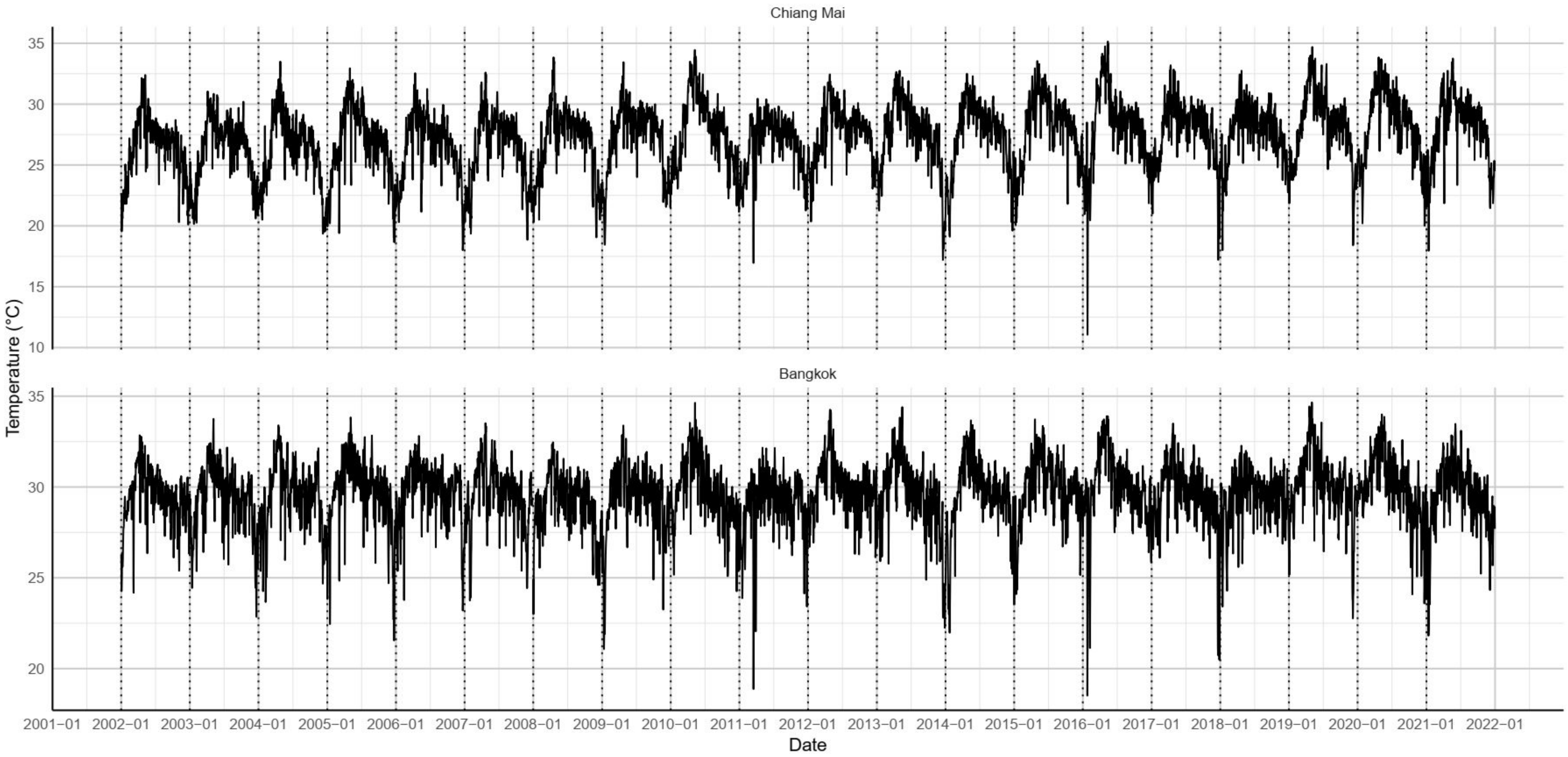


**Figure S2. Time series of daily mean temperature in Chiang Mai and Bangkok.**

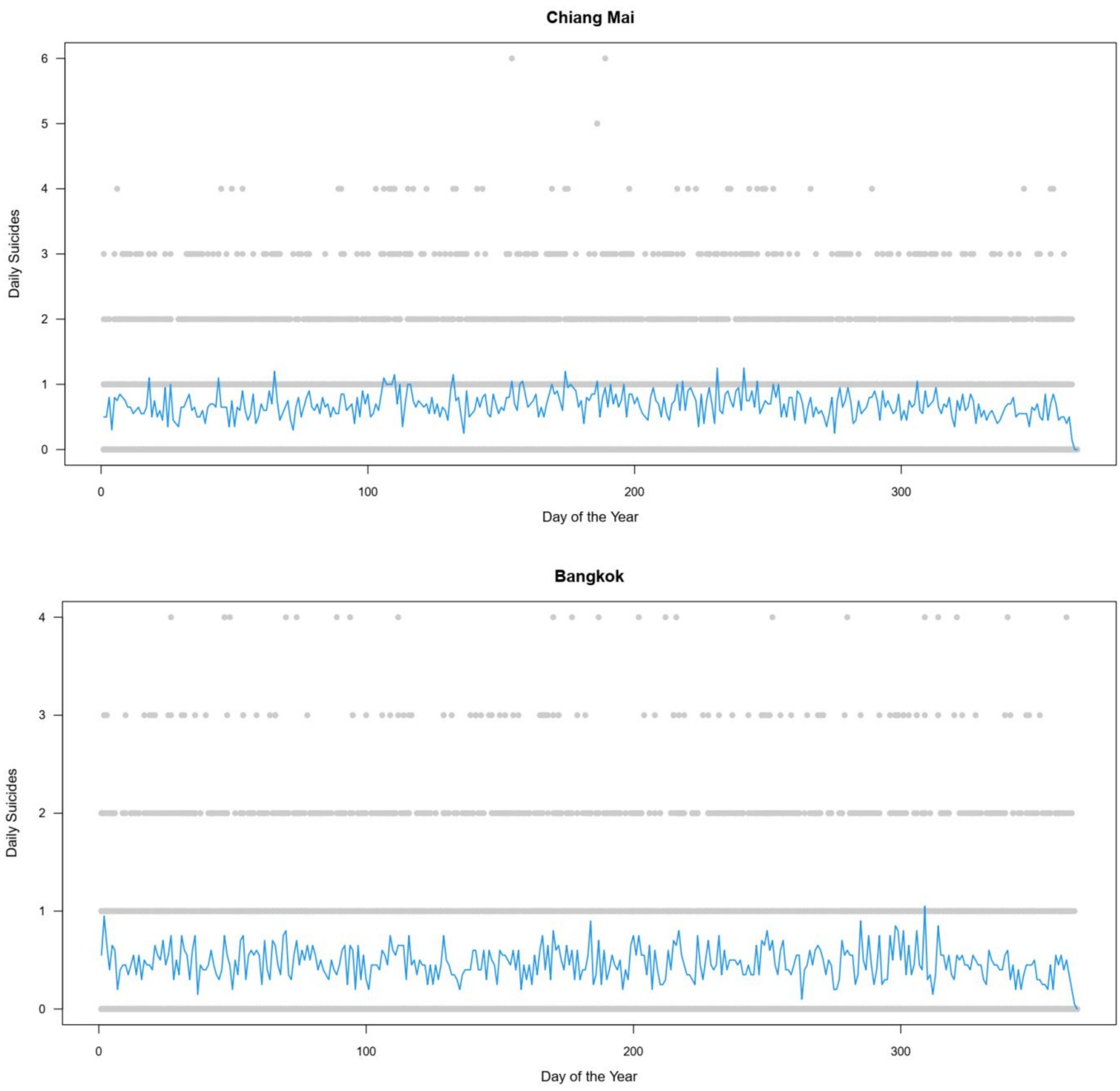


**Figure S3. Seasonal suicide trends in Chiang Mai and Bangkok.** Gray dots correspond to the observed daily suicide counts registered in each day of the year between 2002 and 2021. The blue line depicts the average number of suicides per day of the year.

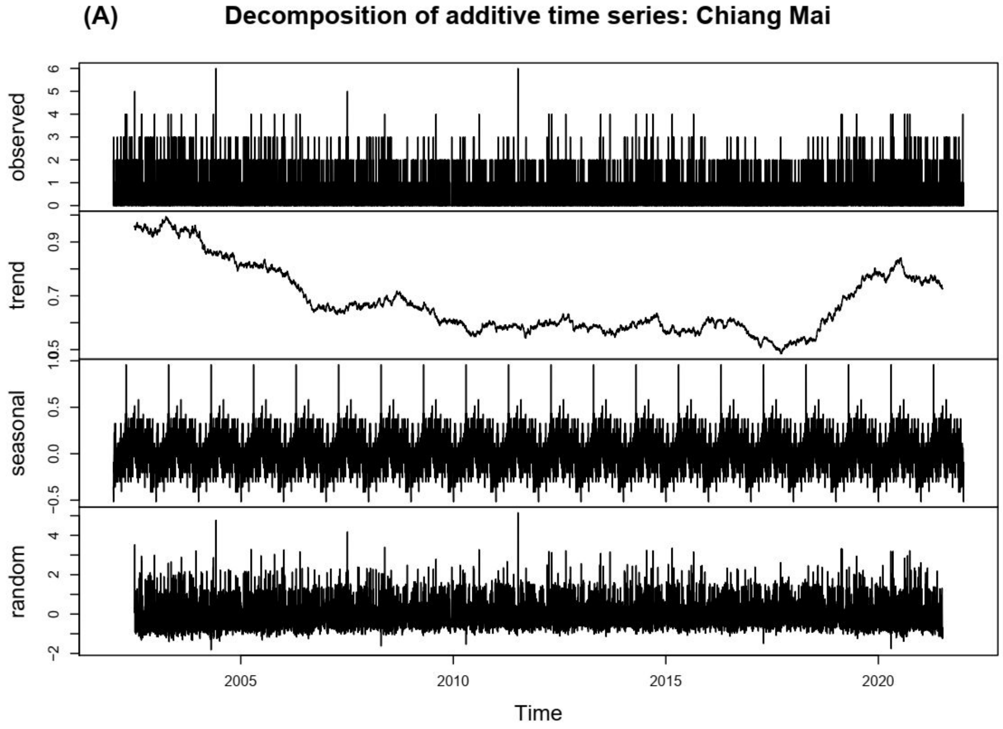


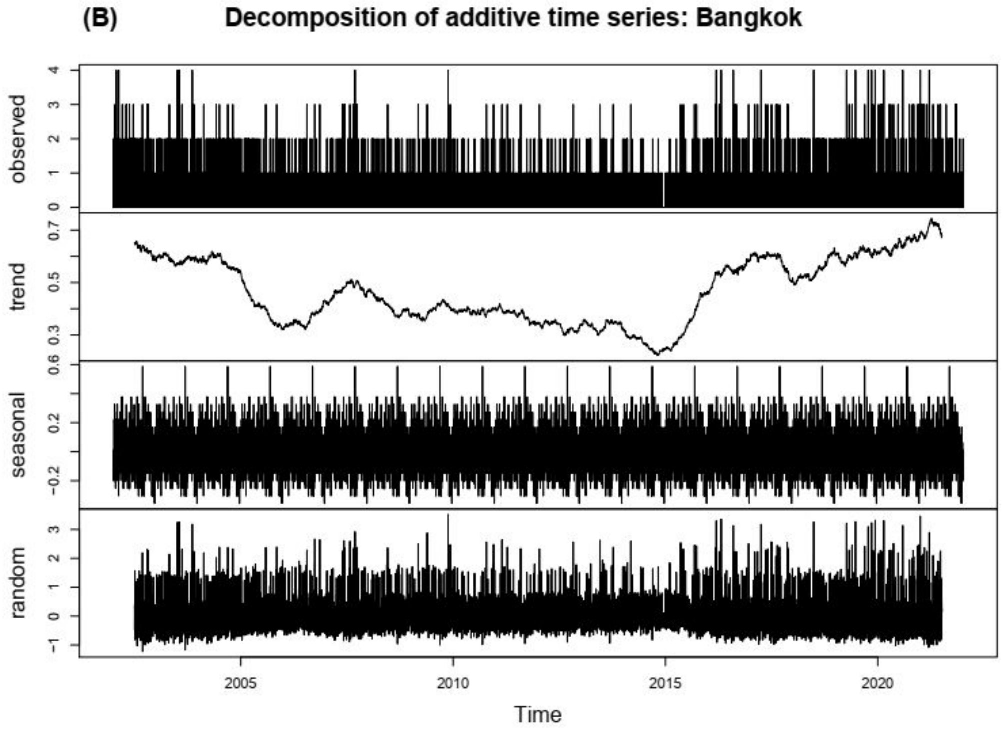


**Figure S4. Decomposition of additive time series of suicide in Chiang Mai and Bangkok.**
(A) Time series decomposition for Chiang Mai.
(B) Time series decomposition for Bangkok.
Each time series is decomposed into four components: observed suicide cases, long-term trend, seasonal trend, and random variation.

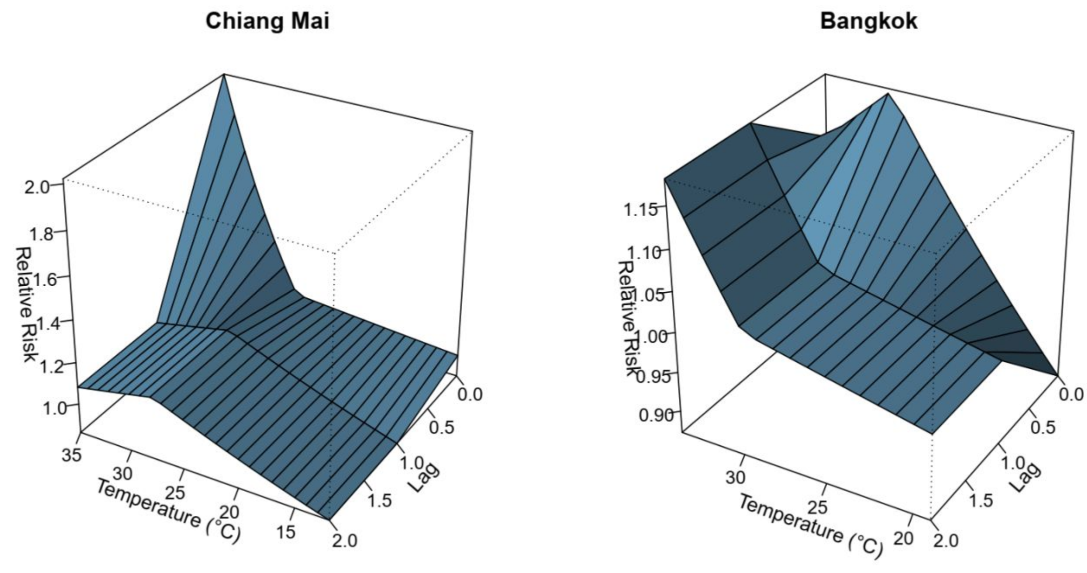


**Figure S5. 3D plot showing the estimated exposure–lag–response association between temperature and suicide in Chiang Mai and Bangkok.**

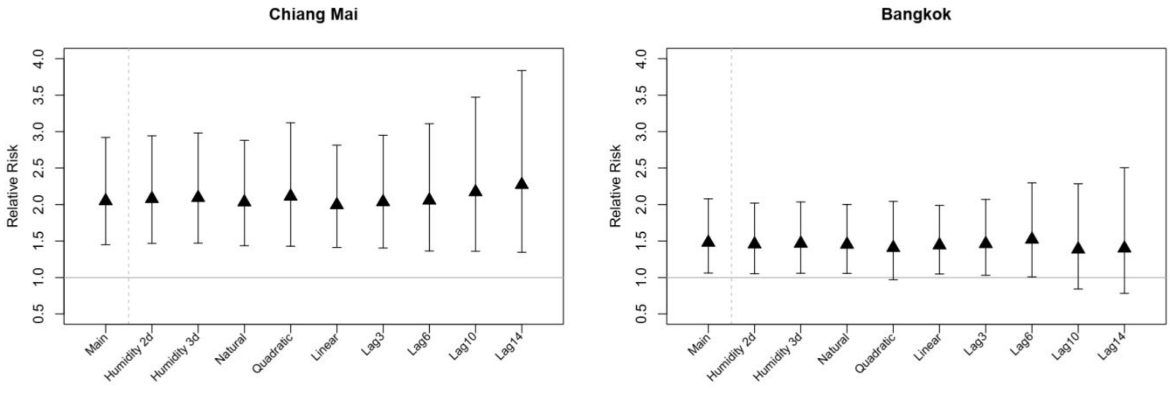


**Figure S6. Sensitivity analysis:** Relative risks with 95% CIs (vertical bars).

Main model and a model that adjusted for the averages of relative humidity (Humidity) as a 2 day moving average and 3 day moving average and models with different parameters such as different spline specification (natural cubic, quadratic b spline, or linear function) and different maximum lag specification (3, 6, 10, or 14).
